# Infectious Pulmonary Artery Pseudoaneurysm Secondary to a Lung Abscess Treated With Pulmonary Artery Coil Embolization: A Case Report

**DOI:** 10.7759/cureus.55762

**Published:** 2024-03-07

**Authors:** Takumi Murakami, Yutaro Otomo, Tatsuya Ito, Kenjiro Sato, Takehiko Ohba

**Affiliations:** 1 Respiratory Medicine, Ome Municipal General Hospital, Tokyo, JPN

**Keywords:** lung abscess, transarterial coil embolization, life-threatening hemoptysis, transcatheter arterial embolization, peripheral pulmonary artery aneurysm, pulmonary artery pseudoaneurysm

## Abstract

Pulmonary artery pseudoaneurysms (PAPs) are uncommon, yet they frequently result in hemoptysis and are associated with a poor prognosis. We report a case of an 87-year-old male patient. Initially, he was admitted to a previous hospital, and diagnosed with a lung abscess in the left lower lobe. On the second hospital day, he developed hemoptysis. A contrast-enhanced chest computed tomography (CT) identified an infectious pulmonary artery pseudoaneurysm. On the ninth hospital day, pulmonary artery coil embolization was successfully performed, significantly improving the patient's condition.

## Introduction

Pulmonary artery pseudoaneurysms (PAPs) are rare vascular anomalies that emerge from various causes, including trauma, iatrogenic injuries such as from Swan-Ganz catheters, or infections [1,2]. PAPs often lead to hemoptysis and have a poor prognosis with potentially fatal outcomes [3]. Surgery and transcatheter arterial embolization (TAE) are the primary treatment options [4,5]. We herein report a case of infectious PAPs secondary to lung abscess, which was successfully treated with pulmonary artery coil embolization.

## Case presentation

An 87-year-old man with hypertension was rushed to his former hospital with a chief complaint of fever and bloody sputum. A plain chest CT revealed an encapsulated fluid collection in the left lower lobe of the lung, and he was admitted to the hospital with a diagnosis of left lower lobe lung abscess. The patient presented a hemoptysis of about 200 mL on the second hospital day and was transferred to our facility on the fourth hospital day.

The patient had a history of esophageal cancer and left renal cancer, both after radical surgery. He had a smoking history with a Brinkman index of 400 and drank alcohol only occasionally.

On admission, he had no fever and percutaneous arterial oxygen saturation (SpO_2_) was 93% on 3 L/min of oxygen via nasal cannula. Physical examination revealed no signs of oral caries, and breath sounds were decreased in the left chest region. The abdominal and neurological examinations were normal.

**Figure 1:**
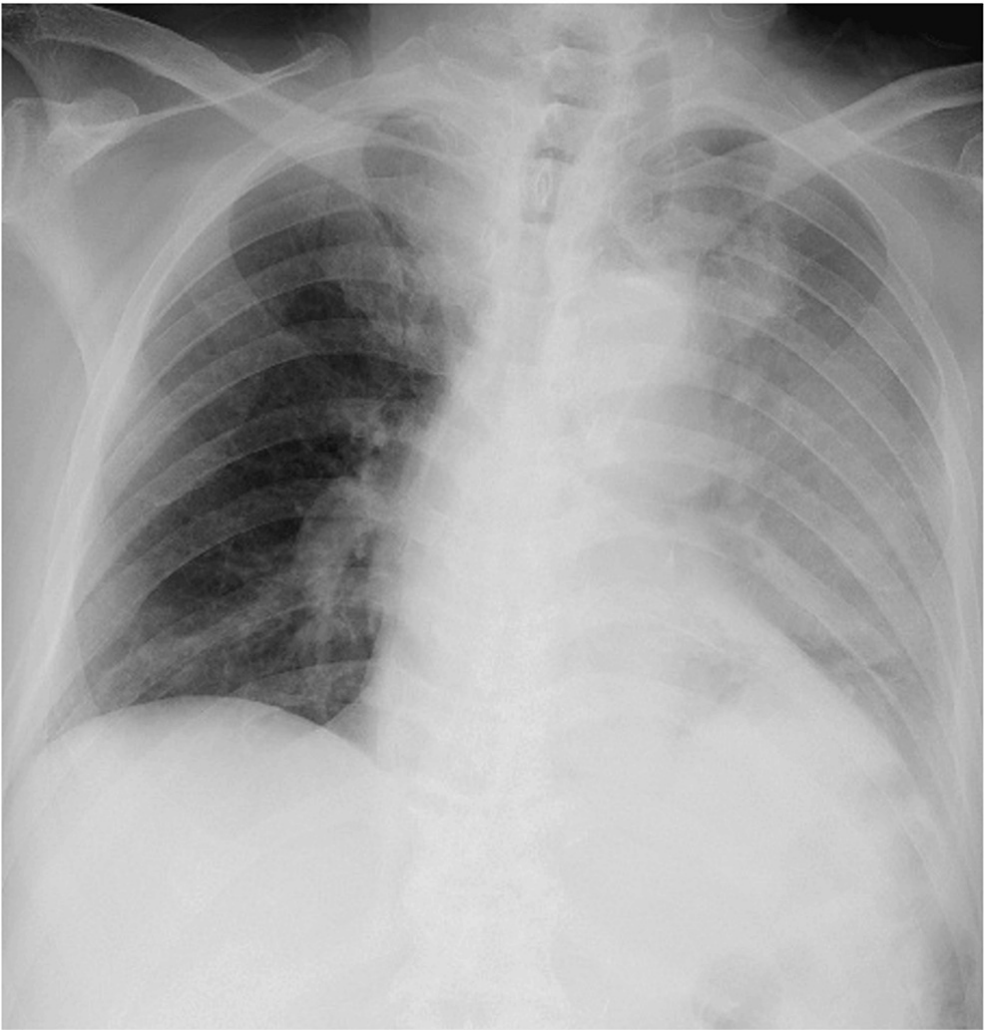
Chest radiography on admission shows an infiltrative shadow in the left lung field.

Blood tests revealed elevated levels of white blood cells (WBC) at 19,090/μL and C-reactive protein (CRP) at 13.91 mg/dL. Blood glucose was 115 g/dL and hemoglobin A1c (HbA1c; National Glycohemoglobin Standardization Program (NGSP)) was 6.2%, indicating no comorbid diabetes. Urinalysis results were normal. A plain chest X-ray showed an infiltrative shadow in the left lower lung field (Figure 1).

**Figure 2:**
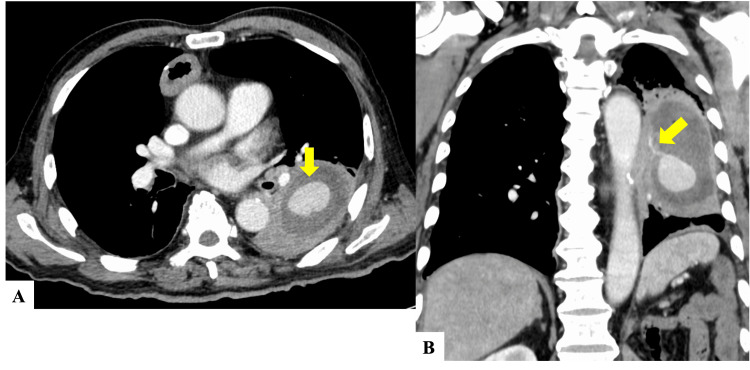
(A) Axial contrast-enhanced chest CT shows an enhanced nodule (PAP) in the lung abscess (arrow). (B) In coronal reconstruction, a vessel feeding into the PAP is observed (arrow). PAP: Pulmonary artery pseudoaneurysm

A contrast-enhanced chest computed tomography (CT) showed an encapsulated fluid collection in the lower lobe of the left lung. Within this collection was a mass measuring 32mm at its longest diameter (Figure 2A). This mass was connected to the left lower pulmonary artery and showed enhancement with the contrasting agent (Figure 2B).

Following a clinical assessment, the patient was diagnosed with a left lower lobe pulmonary abscess and an infectious pulmonary artery pseudoaneurysm. He was initially treated with 13.5 g/day of tazobactam-piperacillin and tranexamic acid. Although massive hemoptysis was absent, a pulmonary angiography was conducted on the ninth hospital day because of a continuous increase in bloody sputum.

**Figure 3:**
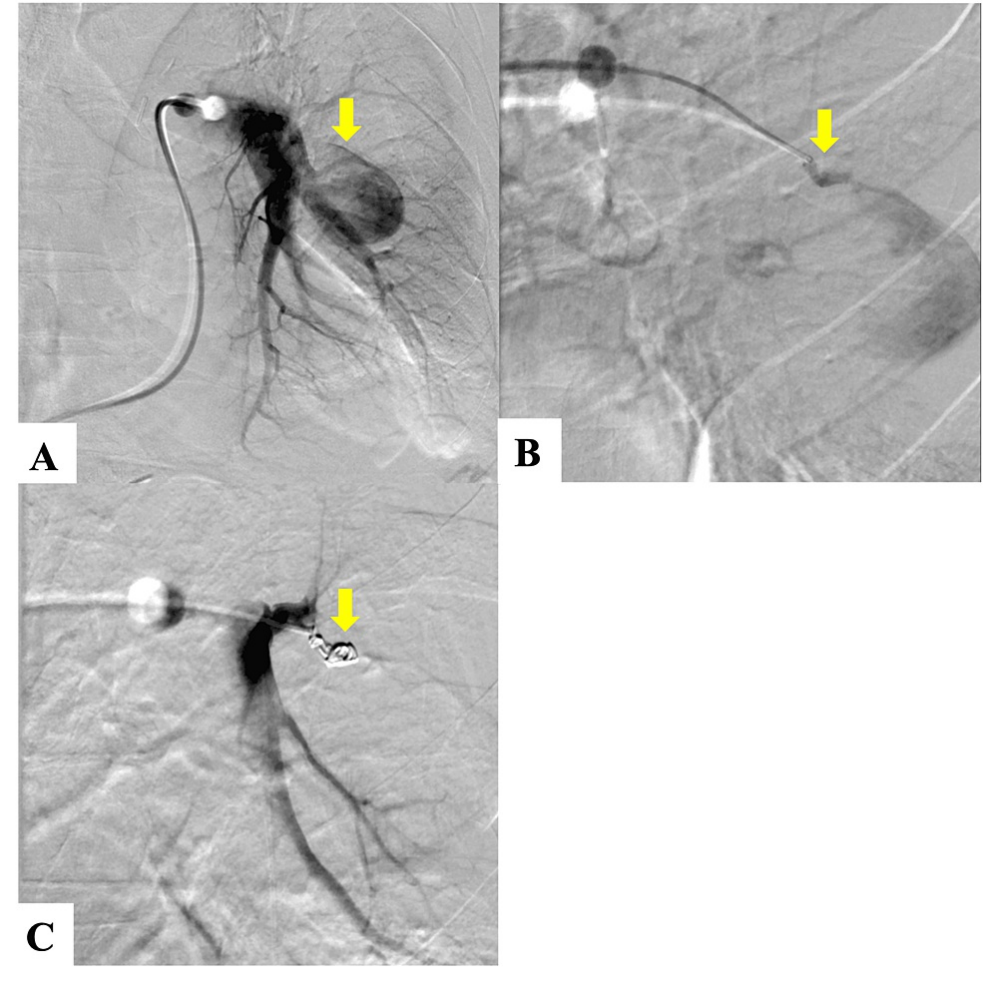
(A) Pulmonary angiography image of the proximal part of a left lower pulmonary artery reveals the PAP (arrow). (B) Selective pulmonary angiography demonstrates the feeding artery to the PAP (arrow). (C) Blood flow disappears after successful transcatheter arterial embolization (arrow). PAP: Pulmonary artery pseudoaneurysm

We identified the feeding artery to the PAP originating from the left A6 segment through repeated selective pulmonary angiography (Figure 3A, 3B). After coil embolization on this artery, aneurysm enhancement disappeared (Figure 3C).

**Figure 4:**
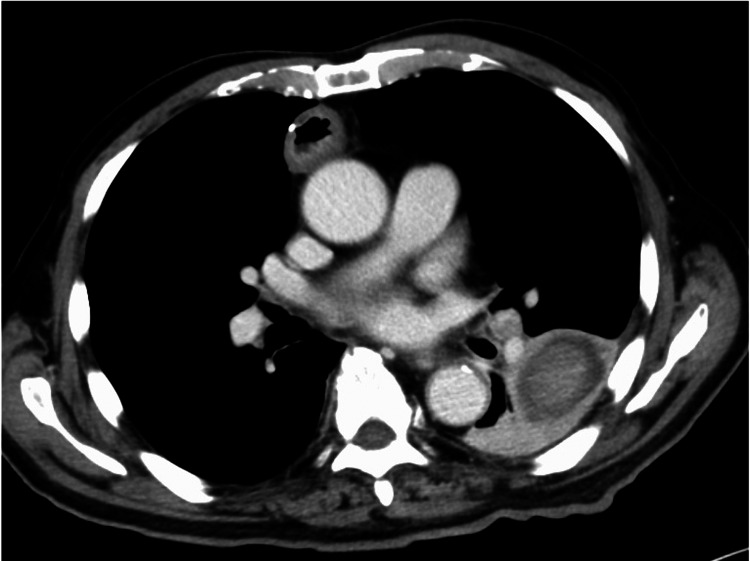
Follow-up chest contrast-enhanced CT reveals a reduction in the abscess size and no enhancement of the PAP. PAP: Pulmonary artery pseudoaneurysm

After the procedure, the bloody sputum disappeared. On the 15th hospital day, a contrast-enhanced chest CT was conducted again, which confirmed a reduction of the lung abscess and the PAP enhancement (Figure 4).

On the 16th hospital day, we switched an antibiotic treatment to oral administration (clavulanic acid 375mg, amoxicillin 1500mg), and the patient continued to recover without any relapse. Finally, he was transferred back to the referring hospital on the 24th hospital day.

## Discussion

PAPs are uncommon vascular anomalies, with previous reports indicating an incidence rate of 0.007% for pulmonary artery aneurysms (PAAs) or PAPs [6]. Specifically focusing on PAPs, a retrospective review at a large teaching hospital reported only 24 cases over 14 years [7].

PAPs are histologically composed of a tunica media and adventitia [8]. Therefore, the risk of rupture is higher compared to PAAs. The mortality rate associated with rupture has been reported to exceed 50%, indicating an extremely poor prognosis [3].

The etiology of PAPs includes trauma, iatrogenic injury from procedures such as Swan-Ganz catheters, airway infections, septic pulmonary embolism, bronchiectasis, and lung cancer [1,2]. While infections including tuberculosis are the primary cause, instances resulting secondarily from lung abscesses are rare, as in our case [9].

Previously, the detection of PAPs primarily relied on pulmonary angiography. Recently, though, CT angiography (CTA) has become the preferred method due to its less-invasive approach [10]. In this case, identification of the feeding artery via CTA was achieved through axial images and multiplanar reconstruction (MPR). However, CTA may fail to detect PAPs in some cases, reaffirming the indispensable role of pulmonary angiography as a diagnostic resource [11].

PAPs were traditionally treated with surgery like lobectomy; however, TAE has recently emerged as the preferred treatment option, owing to its minimally invasive nature [4,5]. Surgical procedures are often performed in refractory cases, such as when hemostasis cannot be achieved through TAE.

In nine case reports of PAPs associated with lung abscesses we reviewed [12-20], treatments included conservative management (two cases), TAE alone (four cases), surgery alone (two cases), and surgery following TAE (one case) (Table 1).

**Table 1:** Summary of PAPs secondary to lung abscesses. M: male, F: female, NR: not reported, TAE: transcatheter arterial embolization, PAPs: pulmonary artery pseudoaneurysms

Reference	Age	Sex	Comorbidities	Hemoptysis	Diameter (mm)	Procedure	Outcome
[12]	63	M	Hypertension	Yes	NR	Surgery (lobectomy)	Alive
[13]	66	M	None	Yes	NR	TAE	Death
[14]	28	M	None	Yes	20	TAE	Alive
[15]	61	F	NR	Yes	18	Surgery (lobectomy)	Alive
[16]	49	F	None	Yes	NR	TAE + Surgery (lobectomy)	Alive
[17]	57	F	NR	Yes	13	Conservative treatment	Alive
[18]	93	M	Aortic stenosis	Yes	NR	TAE	Alive
[19]	21	F	Depression	Yes	6	Conservative treatment	Alive
[20]	58	M	NR	Yes	NR	TAE	Alive
Present case	87	M	Hypertension	Yes	32	TAE	Alive

TAE frequently served as the initial treatment approach, aligning with prior literature; however, surgery was also a prominent treatment option. This reflects the complex management and inherent treatment resistance characteristic of lung abscesses.

We chose TAE as the primary treatment; however, detailed guidelines for pulmonary aneurysms and pulmonary artery pseudoaneurysms remain undefined. Accumulating more case studies and conducting large-scale clinical trials are essential to compare the efficacy of TAE and surgery.

## Conclusions

We encountered a case of infectious PAP secondary to a lung abscess. While PAPs can lead to hemoptysis and often result in a fatal outcome, we successfully treated the patient with pulmonary artery coil embolization. However, no guidelines currently exist for the treatment of PAAs and PAPs. Although further accumulation of case studies is necessary, we consider TAE an effective treatment option under circumstances like those presented in this case.
